# Lower extremity joint kinetics and lumbar curvature during squat and stoop lifting

**DOI:** 10.1186/1471-2474-10-15

**Published:** 2009-02-02

**Authors:** Seonhong Hwang, Youngeun Kim, Youngho Kim

**Affiliations:** 1 Department of Biomedical Engineering, Yonsei University Graduate School, Wonjusi, Gangwon-do, South Korea; 2 Department of Mechanical Engineering, Dankook University, Seoul, South Korea; 3 Department of Biomedical Engineering and Institute of Medical Engineering, Yonsei University, Seoul, South Korea

## Abstract

**Background:**

In this study, kinematics and kinetics of the lower extremity joint and the lumbar lordosis during two different symmetrical lifting techniques(squat and stoop) were examined using the three-dimensional motion analysis.

**Methods:**

Twenty-six young male volunteers were selected for the subjects in this study. While they lifted boxes weighing 5, 10 and 15 kg by both squat and stoop lifting techniques, their motions were captured and analyzed using the 3D motion analysis system which was synchronized with two forceplates and the electromyographic system. Joint kinematics was determined by the forty-three reflective markers which were attached on the anatomical locations based on the VICON Plug-in-Gait marker placement protocol. Joint kinetics was analyzed by using the inverse dynamics. Paired t-test and Kruskal-Wallis test was used to compare the differences of variables between two techniques, and among three different weights. Correlation coefficient was calculated to explain the role of lower limb joint motion in relation to the lumbar lordosis.

**Results:**

There were not significant differences in maximum lumbar joint moments between two techniques. The hip and ankle contributed the most part of the support moment during squat lifting, and the knee flexion moment played an important role in stoop lifting. The hip, ankle and lumbar joints generated power and only the knee joint absorbed power in the squat lifting. The knee and ankle joints absorbed power, the hip and lumbar joints generated power in the stoop lifting. The bi-articular antagonist muscles' co-contraction around the knee joint during the squat lifting and the eccentric co-contraction of the gastrocnemius and the biceps femoris were found important for maintaining the straight leg during the stoop lifting. At the time of lordotic curvature appearance in the squat lifting, there were significant correlations in all three lower extremity joint moments with the lumbar joint. Differently, only the hip moment had significant correlation with the lumbar joint in the stoop lifting.

**Conclusion:**

In conclusion, the knee extension which is prominent kinematics during the squat lifting was produced by the contributions of the kinetic factors from the hip and ankle joints(extensor moment and power generation) and the lumbar extension which is prominent kinematics during the stoop lifting could be produced by the contributions of the knee joint kinetic factors(flexor moment, power absorption, bi-articular muscle function).

## Background

Low back pain(LBP) is a prevalent problem which causes human suffering and cost for workers and their employers. 60~80% of the adult population have experiences of LBP at least once in their lifetimes [1-5]. Despite improved working conditions, including progress due to automation, many objects in the industry are still handled manually. Among basic manual material handling (MMH) activities, lifting has most frequently been associated with LBP[6,7]. Recently, there have been many researches about lifting such as three-dimensional motion analyses, musculoskeletal simulations and medical imaging studies. The most commonly advised lifting technique is the squat technique, in which the knees are flexed [8]. It can easily be understood that compliance with this advice is often low, given the high energetic cost of this technique [9-11]. Van Dieen et al.[12] conducted a comprehensive review on 27 biomechanical studies, comparing stoop and squat techniques, and concluded that no justification existed for advocating squat technique. Jager and Luttman[13] used a three-dimensional dynamic model to estimate lumbar compression and found that compression was barely influenced by lumbar curvature. By observations of physiologic, psychologic, biomechanical and clinical evidence on three lifting techniques; squat, semi-squat, and stoop, Leon Straker[14] reported that all those lifting techniques had both advantages and disadvantages depending on circumstances. Burgess-Limerick[15] presented a general guideline on the method to lift with less damage. Besides, recent studies have shown that many variables exist depending on different lifting methods [16-18].

In this study, lumbar, hip, knee, and ankle joint motions and lumbar spine curvatures during squat and stoop lifting of three different weights were analyzed using the 3-D motion analysis to find out the function of lower limb motions contributing to the lumbar joint.

## Methods

This study was approved by the Institutional Review Board of the Yonsei University and the Wonju Christian Hospital. Twenty-six young male volunteers who had no problems in both lifting and walking were selected as the subjects in this study (Table 1). Informed consent was obtained from all participants.

**Table 1:** Subject information (N = 26)

	**Mean ± S.D**	**range**
Age (year)	23.5 ± 0.76	22 ~24
Weight (kg)	66.5 ± 6.37	55.6 ~74.5
Height (cm)	172.1 ± 6.03	163.4 ~183.5

Two forceplates(Kistler Instrumente AG, Switzerland) and a surface EMG system(MA 300, Motion Lab Systems Inc., USA) were synchronized with the 3D motion analysis system(VICON Motion System Ltd., UK). A total of 31 reflective markers were attached on the anatomical locations according to the VICON Plug-in-Gait marker placement protocol. Besides that, additional four markers(V1~V4) were mounted on the back along the spinous processes to define the spinal curvature. The boxes (34 × 34 × 27.5 cm) weighed 5, 10 and 15 kg, and had the same sized handles. Subjects were asked to lift those boxes using two different techniques (squat and stoop) in their comfortable speed. Joint moments and joint powers in the lower extremities were calculated using the inverse dynamics and the support moment was also determined as the summation of all lower extremity joint moments[19,20].

Paired t-test was used to determine the statistical difference of the maximum lumbar joint moments between the squat and stoop liftings, and the Wilcoxon test was used to compare the maximum joint power among three different weights. The Kruskal-Wallis test was used to compare the joint angles and moments with respect to the increase of weights when the lumbar lordosis appears.

## Results

### Joint angles

**Figure 1:**
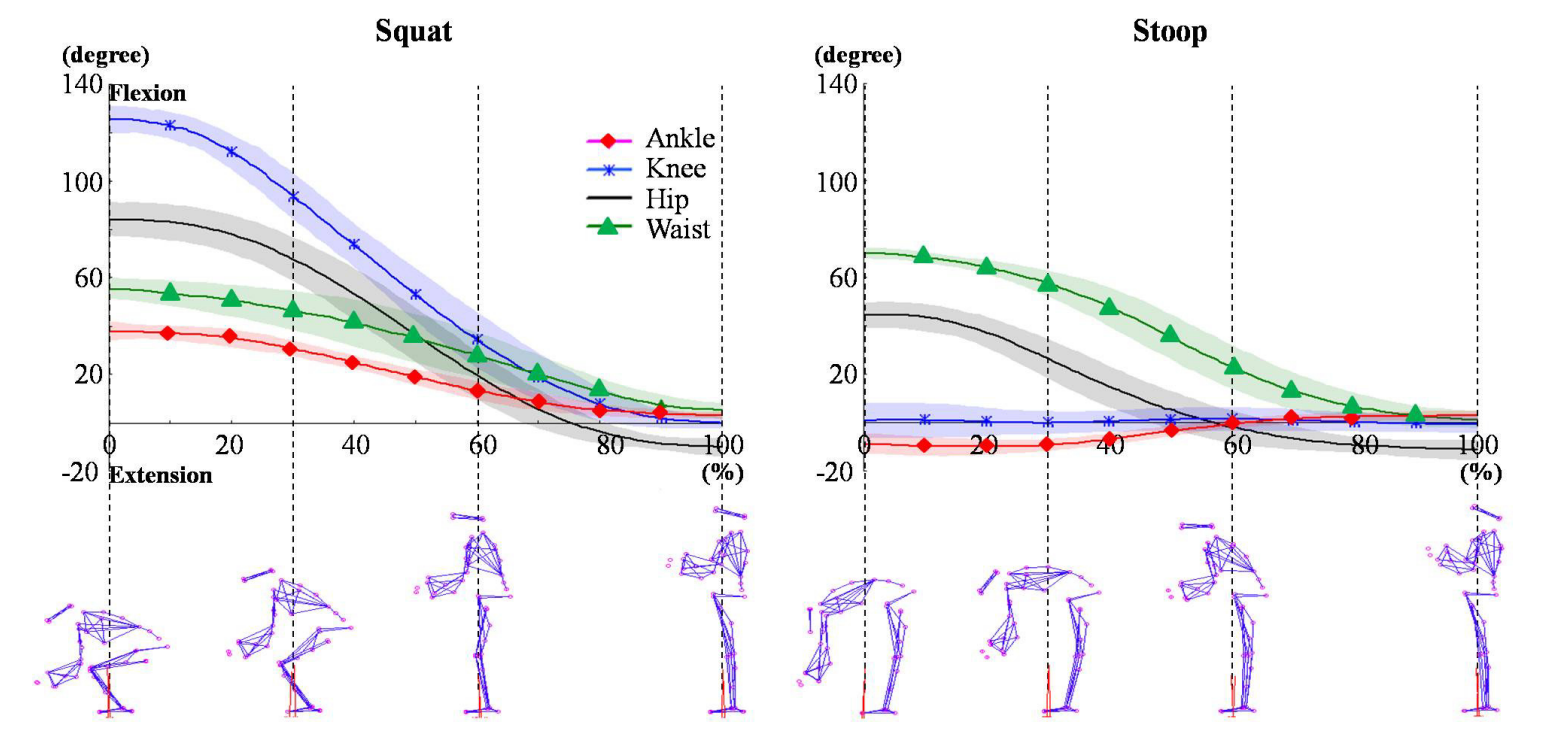
**Joint angles in sagittal plane during squat and stoop lifting**. Joint angles of waist, hip, knee, and ankle were not significant different in different weights (5, 10, and 15 kg), however the range of motion(ROM) of each joint was different between the two lifting methods (squat and stoop).

The subjects lifted the objects as their own comfortable speed and the mean speeds were 0.59 m/s(± 0.14) in squat lifting and 0.60 m/s(± 0.10) in stoop lifting. Figure 1 represents the lower extremity joint angles on the sagittal plane during lifting. Though different weights were lifted, significant differences were not found in the range of motions(ROMs). However, between two techniques, ROMs for the same joints showed significant difference. The knee joint ROM showed the largest difference between two techniques(Figure 1).

### Joint moments

**Figure 2:**
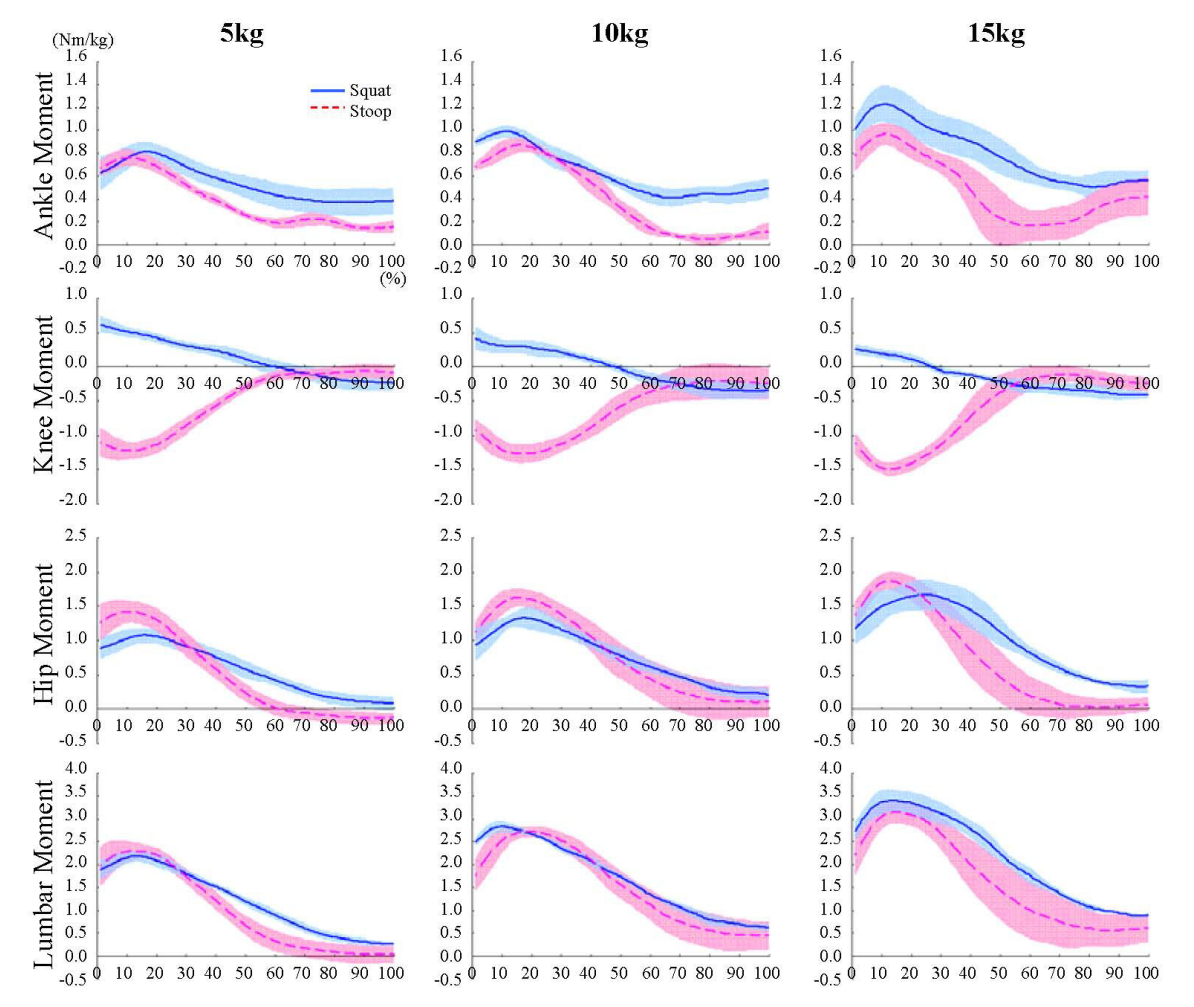
**Joint moments during squat and stoop lifting**. The maximum ankle joint moment was larger in the squat lifting than in the stoop lifting. The knee joint moment was changed from extension moment to flexion moment during the squat lifting, however only the flexion moment was showed during whole period of the stoop lifting. The maximum hip joint moment was larger in the stoop lifting than in the squat lifting. The maximum lumbar joint moment was not different between the squat and stoop lifting significantly.

Joint moments for the different object weights during squat and stoop lifting were plotted in Figure 2. The ankle joint moment was larger in the squat lifting than in the stoop lifting. Only the knee flexion moment existed during the whole process of the stoop lifting. However, in the squat lifting, the knee joint moment changed from extension to the flexion moment, and this turn-over occurred earlier as the object weight increased. The hip extension moment increased to its maximum value as soon as lifting started, and then it decreased to nearly zero. For all weights, the maximum hip extension moment in the stoop lifting was always larger than that in squat. The differences of the maximum lumbar extension moments between the squat and stoop were negligible at 5 and 10 kg. Rather, it was larger in squat than in stoop when 15 kg was lifted (p < 0.05).

### Support moment

**Figure 3:**
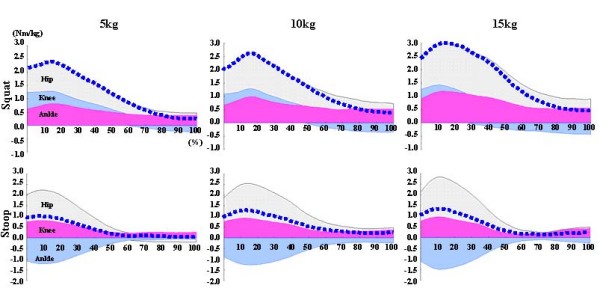
**Support moments during squat and stoop lifting**. Dotted line represents the total support moment and each joint moment is also described. Hip and ankle extension moments contributed to the most part of the support moment during squat lifting, and the knee flexion moment played an important role in the stoop lifting.

Lower extremity joint moment could be analyzed with the concept of 'support moment'[15]. Figure 3 shows that the contributions of each lower extremity joint for the support moment in two different lifting techniques. The dashed line in the figure represents the total support moment during lifting, and the height between two curves at any time represents the contribution to the support moment of that joint. At the initial stage of lifting, the hip and ankle joint extension moments were large during the squat lifting. On the other hand, during stoop lifting (the knee joint ROM was nearly zero), there were large knee flexion moments at the initial stage of lifting. Total support moments were larger in squat than in stoop because of the negative values of the knee moment in stoop lifting. Therefore, hip and ankle contributed to the most part of the support moment during squat lifting, and the knee flexion moment played an important role in the stoop lifting.

### Joint power

**Figure 4:**
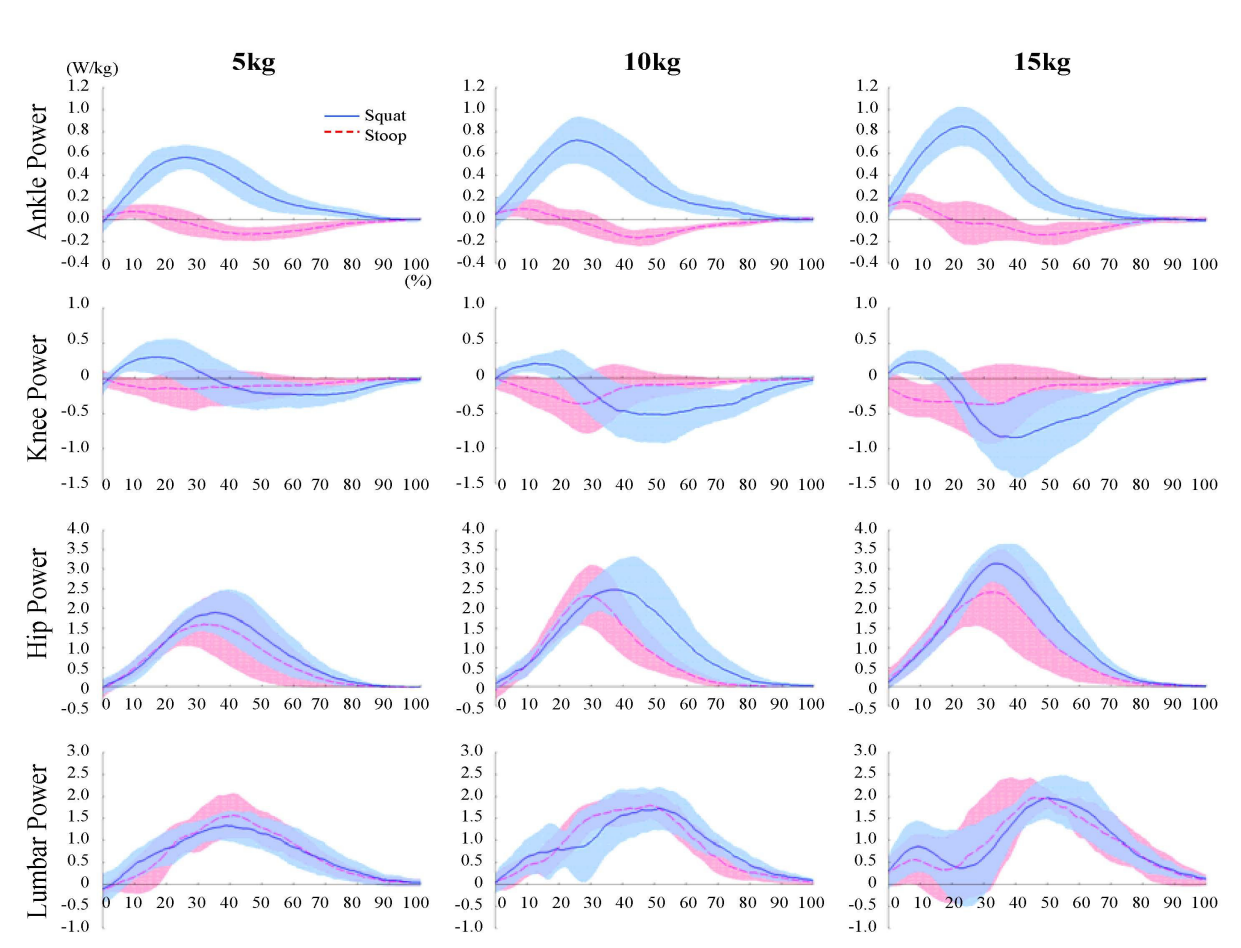
**Joint powers during squat and stoop lifting**. The ankle, hip, lumbar joints generated power(concentric contraction) but only the knee joint absorbed power(eccentric contraction) for the most part during squat lifting. The hip and lumbar joint generated power(concentric contraction) in contrast with the ankle and knee joint which absorbed power(eccentric contraction) for the most part.

Figure 4 shows the joint power for different lifting weights during the squat and stoop lifting. In the squat lifting, the ankle, hip, lumbar joints generated power (concentric contraction) but only the knee joint absorbed power (eccentric contraction) for the most part, except for the initial stage in which the power was generated slightly. In addition, the quantities of generated knee power decreased as the object weight increased.

In the stoop lifting, the hip and lumbar joint generated power (concentric contraction) in contrast with the ankle and knee joint which absorbed power (eccentric contraction) for the most part.

### Dynamic EMG

**Figure 5:**
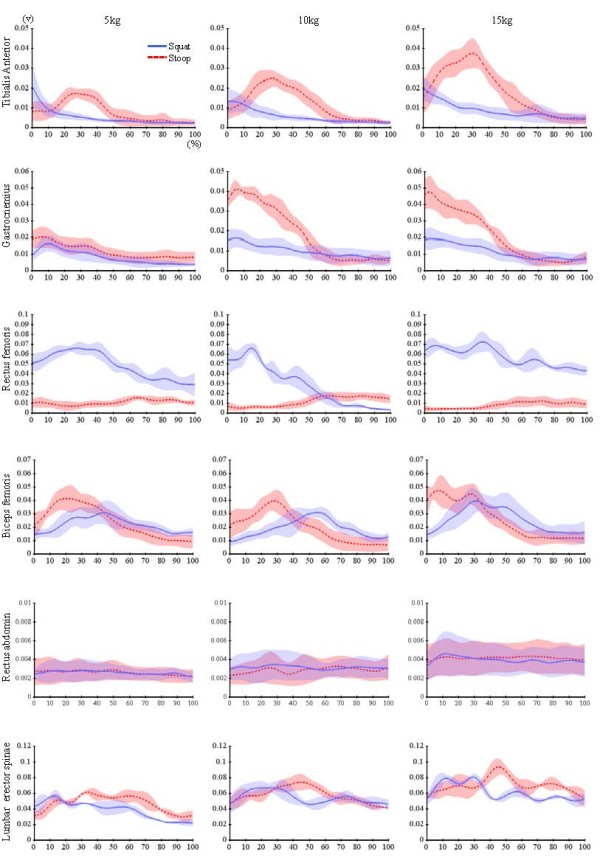
**Linear enveloped dynamic EMGs of lower limb muscles during squat and stoop lifting**. Biceps femoris and rectus femoris showed large variances of activation during the squat lifting. Tibialis anterior, medial gastrocnemius, and biceps femoris showed large variances of activation during the stoop lifting.

Biceps femoris and rectus femoris showed large variances of activation during the squat lifting. Tibialis anterior, medial gastrocnemius, and biceps femoris showed large variances of activation during the stoop lifting (Figure 5). Rectus abdominis and lumbar erector spinae had not significant differences between the squat and stoop lifting.

Co-contraction of the bi-articular knee antagonists (rectus femoris and biceps femoris) were observed markedly during the squat lifting. The concentric contraction of the tibialis anterior and the simultaneous eccentric contraction of the gastrocnemius during the stoop lifting also observed in the ankle joint.

### Lumbar curvature

**Figure 6:**
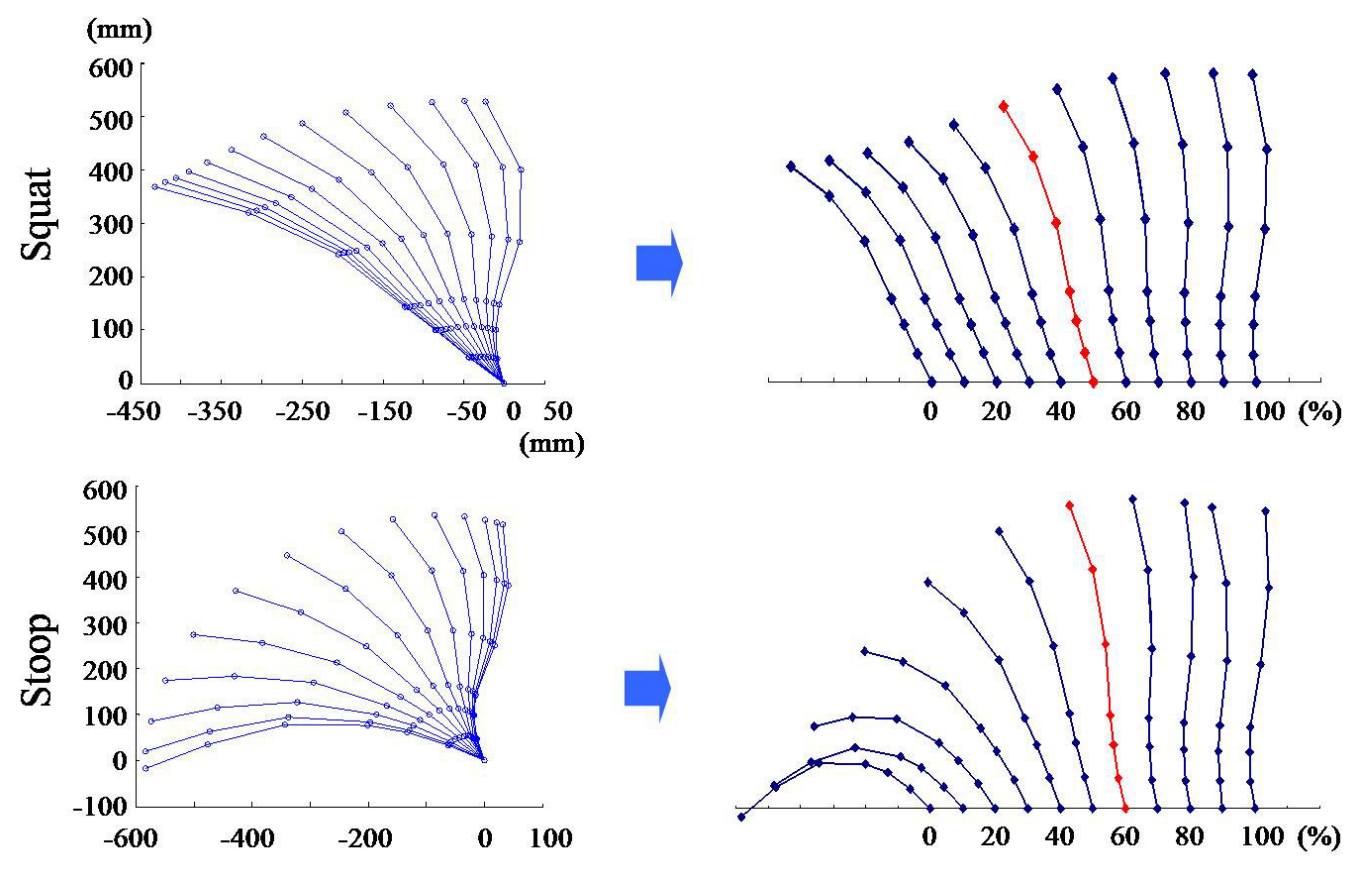
**Lumbar spine curvatures during squat and stoop lifting**. Lumbar curvature was changed from the kyphosis to the lordosis about 50% in the squat lifting, and 60% in the stoop lifting regardless of weights.

Figure 6 represents the spine curvature when the lumbar lordosis appeared. Lumbar curvature was changed from the kyphosis to the lordosis about 50% in the squat lifting, and 60% in the stoop lifting regardless of weights. Lower limb joint angles and moments at that time were showed at Table 2, and its difference among the three different object weights were tested by the nonparametric central tendency test in the three groups (5, 10, 15 kg). The knee angle, the ankle angle, the lumbar moment had significant differences as the weight increased in the squat lifting. The lumbar angle, the lumbar moment and the hip moment had significant differences as the weight increased (p < 0.05) in the stoop lifting. Table 3 shows the correlation coefficients between the "lumbar" and the "lower extremities", comparative parameters were the angles and moments. The knee angle in the squat, the hip and ankle angles in the stoop showed strong correlation with the lumbar angle. All three joint moments(hip, knee, ankle) showed the correlations with the lumbar moment in the squat lifting, however only the hip moment had the correlation with the lumbar moment in the stoop lifting (p < 0.01).

**Table 2:** Joint angles and moments when the lumbar lordosis appears during lifting (Kruskal Wallis)

Mean (SD)		Squat lifting			Stoop lifting		
		5 kg	10 kg	15 kg	5 kg	10 kg	15 kg
Joint angles (deg)	Lumbar joint	31.32(3.41)	31.14(4.05)	28.01(3.71)	28.19(1.96)*	25.96(2.14)*	24.79(4.53)*
	Hip joint	18.23(22.16)	13.55(20.55)	5.67(15.49)	-1.90(5.11)	-1.89(6.23)	-6.05(4.79)
	Knee joint	23.06(18.41)*	18.65(13.54)*	8.15(6.30)*	-0.83(3.95)	-1.18(2.81)	-2.15(2.38)
	Ankle joint	10.28(6.71)*	7.32(4.71)*	2.80(2.28)*	1.25(2.58)	0.09(2.12)	-0.25(2.93)

Joint moments (Nm/kg)	Lumbar joint	0.89(0.33)*	1.17(0.33)*	1.19(0.21)*	0.60(0.09)*	0.85(0.12)*	0.97(0.09)*
	Hip joint	0.51(0.26)	0.62(0.28)	0.61(0.20)	0.32(0.07)*	0.42(0.10)*	0.46(0.11)*
	Knee joint	-0.20(0.10)	-0.36(0.14)	-0.47(0.11)	-0.38(0.13)	-0.43(0.12)	-0.48(0.11)
	Ankle joint	0.48(0.20)	0.57(0.20)	0.54(0.15)	0.42(0.17)	0.43(0.18)	0.45(0.24)

*: There is significant difference in angle or moment with respect to the weight increase (p < .05)

**Table 3:** Correlation coefficients between lumbar and lower extremity joint

Correlation coefficient (p)		Squat lifting		Stoop lifting	
		Lumbar angle	Lumbar moment	Lumbar angle	Lumbar moment
Lower limb joint angle (deg)	Hip angle	0.219(0.398)	-0.334(0.190)	0.822(0.000)*	0.044(0.864)
	Knee angle	0.653(0.005)*	0.018(0.947)	0.375(0.126)	0.525(0.025)
	Ankle angle	0.148(0.571)	-0.295(0.250)	-0.750(0.000)*	-0.280(0.261)

Lower limb joint moment (Nm/kg)	Hip moment	0.381(0.131)	0.975(0.000)*	0.287(0.248)	0.875(0.000)*
	Knee moment	0.352(0.166)	-0.621(0.008)*	0.672(0.002)*	-0.116(0.646)
	Ankle moment	-0.354(0.163)	0.668(0.003)*	-0.802(0.000)*	-0.199(0.428)

*: The correlation coefficient has a statistical significance at p < 0 .01

## Discussion

Without limitation of the assumption of biomechanical model used for calculation of kinematic and kinetic results[21], the limitations of this study were summarized as follows. We just analyzed the representative two lifting techniques on the assumption that they were symmetrical movements. In addition, the movements in the coronal/horizontal plane were not included in this study. Under the in-vitro examination, it was inevitable to keep the subject's motion under control – initial foot position, upper extremity position, knee flexion angle(during squat lifting). The objects were not placed close enough to the body because the reflective markers could be hidden. In fact, lumbar could be often damaged mechanically due to the asymmetric or unbalanced lifting movement.

The heavy weight of object is also critical factor to the lumbar damage but 15 kg was assumed as heavy weight in this study for the safety of the subjects.

The result of the maximum lumbar joint moment comparison between the squat and stoop lifting corresponded to the previous study that there was no conclusive evidence for advocating the squat lifting[12].

The support moment calculated by the summation of the extension moments in the previous study[9], however all moments including flexion moments were summated for support moment in this study because the knee flexors could act as supporters.

It was expected that the joint moment results could be supported by the EMG results. However, the normalized EMG data had large variation among subjects and a lot of data excluded for analysis because of its failure of detection therefore we were focused on the activation patterns to interpret EMG data.

The lumbar lordosis appearance time was important during the lifting motion [22-25], thus we tried to find out the contributions of the lower extremities in relation to the lumbar joint.

The correlation coefficients between the lumbar and the lower extremity were investigated which were calculated by using the angles and moments at the time of lumbar lordosis appearance. The knee angle had the strong correlation with the lumbar angle in the squat lifting, and the hip and ankle angle had the correlation with the lumbar angle in the stoop lifting. These results showed representative kinematic characteristics of each lifting technique. All three lower extremity joint moments had the correlation with the lumbar joint in the squat lifting, and only the hip joint moment had the correlation with the lumbar joint in the stoop lifting.

There are three important bi-articular muscles in the lower body (rectus femoris, biceps femoris, gastrocnemius), and they affects two joints simultaneously [26-30].

In addition, the squat lifting as well as the stoop lifting is the typical closed kinetic chain motion [27-29] so that the bi-articular muscle function is more complex(Lombard paradox:[31,32]). The EMG analysis and the calculation of individual muscle force change using simulation software could be helpful to determine these bi-articular muscle functions.

## Conclusion

1) There were not significant differences in maximum lumbar joint moments between two techniques. Rather, the maximum lumbar extension moment was larger in squat than in stoop when 15 kg was lifted (p < 0.05). This result advocates the previous study.

2) The hip and ankle joint contributed to the most part of the support moment during the squat lifting, and the knee flexion moment played an important role in the stoop lifting.

3) The ankle, hip and lumbar joints generated power and only the knee joint absorbed power in the squat lifting. The ankle and knee joints absorbed power and the hip and lumbar joints generated power in the stoop lifting.

4) The EMG results summarized that the co-contraction of the antagonists was observed markedly in the both lifting techniques; the tibialis anterior and the gastrocnemius in the ankle joint, the rectus femoris and the biceps femoris in the knee joint.

5) At the time of lordotic curvature appearance in the squat lifting, strong correlations were found in all three lower extremity joint moments with the lumbar joint. On the other hand, in the stoop lifting, strong correlations existed in the hip moment with the lumbar joint.

In conclusion, considering the correlation with the lumbar joint, the kinetic factors generated by the ankle and hip joints (the extensor moment and the power generation) mostly lead the knee extension which is the remarkable kinematics in the squat lifting. The lumbar joint's kinematics (ROM) was the largest in stoop lifting. However, this movement couldn't be done safely without the knee joint's kinetic factors (the flexor moment, the antagonistic co-contraction of bi-articular muscles and the power absorption).

## Competing interests

The authors declare that they have no competing interests.

## Authors' contributions

YEK jointly conceived the study with YHK SHH designed and implemented the experiments, acquired and analyzed data, and prepared the manuscript. YEK performed signal processing, and gave technical support. YHK carried out biomechanical interpretation of the complex data. YEK and YHK revised manuscript.

## Pre-publication history

The pre-publication history for this paper can be accessed here:


